# Can mental health competence reduce the higher risk of smoking initiation among teenagers with parents who smoke?

**DOI:** 10.1093/eurpub/ckab102

**Published:** 2021-09-18

**Authors:** Anna Pearce, Emeline Rougeaux, Jessica Deighton, Russell M Viner, Catherine Law, Steven Hope

**Affiliations:** 1 MRC/CSO Social and Public Health Sciences Unit, University of Glasgow, Glasgow, UK; 2 Population, Policy and Practice Research and Teaching Department, UCL Great Ormond Street Institute of Child Health, London, UK; 3 Evidence Based Practice Unit, UCL and the Anna Freud Centre, London, UK

## Abstract

**Background:**

Parental smoking strongly influences adolescent smoking, yet few studies have examined factors that may protect against this. We investigated whether skills-based components of positive mental health (‘mental health competence’, MHC) modified the association between parental and teenager smoking, in the UK-representative Millennium Cohort Study (approximately 18 000 children, born 2000–02; analytic sample: *n *=* *10 133).

**Methods:**

Cohort members (CMs) reported at 14 years (y) whether they had ever smoked cigarettes. A dichotomized variable indicated whether one/both parents smoked when CMs were 11 y. A four-class latent MHC measure captured learning skills and prosocial behaviours at 11 y: High, High–Moderate, Moderate, Low. We examined effect measure modification (on the additive scale) by comparing risk differences (RDs) for CM smoking according to parental smoking, within each MHC class. We then estimated RDs for CM smoking according to combinations of parental smoking and MHC. Analyses accounted for confounding, sample design, attrition and item missingness.

**Results:**

CMs were more likely to smoke cigarettes if their parent(s) smoked (27%) than CMs with no parent(s) who smoked (11%; RD: 16%). When stratified by MHC, RDs were stronger for low MHC (21%; 95% CI 11–31%) than other MHC classes (ranging: 7–11%). Compared to CMs with high MHC and non-smoker parents, those with low MHC and parent(s) who smoked had an RD of 28% (95% CI 20–36%). This was greater than the sum of RDs for those with low MHC and non-smoker parent(s) [7% (2–14%)] plus those with high MHC and whose parent(s) smoked [11% (7–15%)]. There was limited effect measure modification by moderate or High–Moderate MHC.

**Conclusion:**

Improving MHC to moderate levels may help reduce intergenerational transference of smoking.

## Introduction

### Rationale

Worldwide, an estimated 6 million deaths occur each year due to tobacco use.^1^ In the UK, an estimated 7.6 million (16%) adults were current smokers in 2016,^2^ and in the same year more than half a million hospital admissions and 100 000 preventable deaths were attributed to smoking, costing the economy £11 billion.^3^ Despite decreases in smoking rates in high-income countries, around one in four adults in the European region are forecast to be smokers in 2025.^1^ Furthermore, smoking rates are higher in less advantaged groups, and tobacco use is responsible for half of the 9-year (y) difference in life expectancy between the most and least deprived areas of the UK.^4^

Adolescence is a crucial period for the development of health-risk behaviours, such as smoking, and a time of particular vulnerability to the addictive properties of nicotine.^5^ A European Union-wide study of ever smokers under the age of 40 y found that two-thirds commenced smoking before the age of 18 y, and one-third by the age of 15 y.^6^ In the UK, 80% of 50-y-old daily smokers started smoking before the age of 20 y, with similar patterns seen internationally.^7^ In 2015, it was estimated that one in four 15- to 16-y-olds in the European Union had smoked in the last month.^8^ Preventing smoking initiation in adolescence is, therefore, an important part of any tobacco reduction policy, and in 2017 the English Department of Health announced a target that no >3% of young people would smoke regularly by 2022.^3^ Similar policy approaches are being taken internationally.^9^

Smoking initiation in adolescence is influenced by the smoking habits of parents, siblings and other household members, through shaping family rules, modelled behaviours and the availability of tobacco products. In an international meta-analysis, Leonardi-Bee et al.^10^ estimated that 17% of smoking incidences in 15-y-olds can be attributed to the smoking habits of household members. Given this, the authors conclude that the focus of tobacco policy should move beyond smoke-free homes to smoker-free homes.^10^ A complementary preventative approach might be to identify factors that could potentially buffer against the influence of family members who smoke. One such factor is positive mental health.

Positive mental health has been linked to healthy physical and mental development across the life course.^11^ Although the definition of positive mental health varies across disciplines and cultures, it is thought to include not only feelings of well-being (e.g. positive affect and life satisfaction) but also skills (such as basic cognitive and social competencies and emotional regulation),^12^^,^^13^ which are a focus of early childhood education and care frameworks.^14^^,^^15^ A recent conceptualization of positive mental health for children, known as mental health competence (MHC), has been constructed from questions included in existing surveys and administrative data in Australia^16^^,^^17^ and the UK.^18^ MHC focusses on skills-based attributes such as social competence, helping behaviours, responsibility and respect, approaches to learning, and readiness to explore new things. Findings from the UK indicate that high levels of MHC (at the end of primary school) are associated with fewer health-risk behaviours in adolescence, including smoking.^19^ Other research has demonstrated associations between particular MHC skills (including academic ability, self-regulation and executive function) in young people and lower risks of smoking.^20–24^ Furthermore, trial evidence indicates that MHC skills, such as self-regulation and social skills, can be improved through interventions, including in school settings, and that these interventions have short–medium benefits for educational outcomes and health-risk behaviours such as substance misuse.^25^^,^^26^ However, what is not yet known is the extent to which MHC may protect against risk factors for smoking initiation, such as having a parent who smokes.

Our aim was to examine whether MHC modifies the association between parent and teenager smoking. Specifically, we hypothesized that the number of smoking initiation cases linked to exposure to parental smoking would be greater for young people with lower levels of MHC.

## Methods

### Sample

We used data from the UK Millennium Cohort Study (MCS), a longitudinal study of children born in the UK between September 2000 and January 2002.^27^ Families were selected through Child Benefit Records, and a disproportionately stratified clustered sampling design was used to over-represent children living in Wales, Scotland and Northern Ireland, disadvantaged areas, and, in England, areas with high proportions of ethnic minority groups. About 18 818 infants (18 296 singletons) were enrolled in the study. Interviews were carried out by trained interviewers in the home with the main respondent (usually the mother) when cohort members (CMs) were 9 months (*n *=* *18 296), 3 (when an additional 685 singletons were recruited; *n *=* *15 381), 5 y (*n *=* *15 041), 7 (*n *=* *13 681), 11 y (*n *=* *13 112) and 14 y (*n *=* *11 576) of age. As with most longitudinal surveys, respondents from less advantaged backgrounds were more likely to be lost to follow-up, with weights available to account for this.^28^ CMs also completed questionnaires at 7–14 y. Data were downloaded from the UK Data Service, University of Essex and the University of Manchester in May 2017. Ethics approval was granted for each main MCS survey.^29^

We analyzed data on 10 133 CMs who had a measure of MHC at 11 y and who were present at the 14 y survey. Survey weights accounted for sample design and attrition; item missingness was addressed through multiple imputations.

### Measures

#### Exposure: smoking among parents

A dichotomized variable, referred to as *parental smoking* hereafter, indicated whether at least one main carer(s) (predominantly the mother, and father/mother’s partner where present) reported currently using tobacco products (>97% smoked cigarettes or hand-rolled tobacco, referred to as ‘smoking’ hereafter) when the CM was 11 y.

#### Outcomes: CM smoking (cigarette and e-cigarette)

Cigarette smoking status at 14 y indicated whether the CM had never smoked versus ever smoked a cigarette (combined: had tried/used to smoke/currently smoked).

Smoking e-cigarettes (at 14 y), indicated whether the CM had never versus ever smoked an e-cigarette.

#### Effect modifier: mental health competence

MHC at 11 y was measured using a latent variable described elsewhere,^18^ representing important aspects of prosocial behaviours and learning skills. It was derived from eight positively worded items from the parent-reported Strengths and Difficulties Questionnaire (SDQ)^30^: all five items from the prosocial behaviours scale (considerate, shares, helpful, kind and volunteers) represented pro-social behaviours while learning behaviours were represented by two items from the hyperactivity scale (thinks things through and completes tasks) and one item from the conduct problems scale (obedient). MHC consisted of four classes: CMs with high prosocial behaviours and learning skills (‘High MHC’), high prosocial behaviours and moderate learning skills (‘High–Moderate MHC’), moderate skills for both (‘Moderate MHC’) and moderate prosocial behaviours but low learning skills (‘Low MHC’).

#### Confounding

We adjusted for several baseline confounders (i.e. factors that might influence the exposure and the effect modifier or outcomes): CM’s ethnicity [due to small numbers in some categories, a binary measure was used: White; Other (Mixed, Black British, Indian, Pakistani and Bangladeshi, Other)] and mother’s highest academic qualification [dichotomized as General Certificate in Secondary Education (GCSE) grades A*–C (or equivalent) or above, versus GCSE Grades D–G (or equivalent) or below], measured when the cohort child was age 9 months (or 3 y if not available in infancy), as these were either fixed or relatively stable over time. Measures of mother’s psychological distress (Kessler-6 scale, dichotomized as none-low and Moderate–High distress), socio-emotional problems in the CM (using total difficulties SDQ, dichotomized as normal and borderline-abnormal using validated cut-offs), and family structure (natural couple, reconstituted and lone parent family) were adjusted for at 7 y (thus preceding measurement of the exposure, to minimize the potential impact of reverse causality). Equivalized household income (continuous) was measured at 11 y (same age as the exposure), as income is not stable over time and the potential for reverse causality was minimal (i.e. income was unlikely to be influenced by MHC). In addition, we adjusted for CM’s sex, as a potential confounder of the association between MHC and smoking. The hypothesized relationships between the main variables of interest are summarized in figure A1 (Supplementary Appendix SA1).

### Analysis

We examined the association between parental and CM smoking by calculating risk differences (absolute differences in smoking prevalence between parental smoking groups, RDs) using linear regression, which accurately estimates RDs when modelling binary outcomes,^31^ with robust standard errors. Thus, we examined interactions on the additive scale, which are of greatest relevance for public health purposes, because they indicate where the largest number of cases may be prevented through intervening on the modifier.

CM cigarette smoking and e-cigarette smoking were examined as separate outcomes. All analyses were carried out before and after adjustment for potential confounding. In order to examine whether MHC might buffer against the potential risk of parental smoking on CM’s smoking, we adopted an effect measure modification approach (referred to as effect modification hereafter). Specifically, we hypothesized that the elevated risk of smoking among CMs who had at least one parent who smoked would be stronger for those with lower MHC. Adopting a framework recommended in the Strengthening the Reporting of Observational Studies in Epidemiology guidance^32^ and Knol and VanderWeele,^33^ we examined effect modification using two approaches (which are statistically equivalent but present the results in different ways).

Approach 1: we examined whether the association between parent and CM smoking varied by MHC. Specifically, we estimated RDs, representing the absolute difference in CM smoking prevalence between those who did and did not have at least one parent who smoked, within each MHC class. Measures of effect modification on the additive scale represent the size of the absolute difference between the RDs for CM smoking by parent(s) smoking, within the Moderate–High, Moderate and Low MHC groups, compared with the baseline (High MHC). A measure greater (or less) than zero indicates the presence of a positive (or negative) additive interaction.

Approach 2: we estimated RDs for CM smoking according to the combination of parental smoking and MHC (baseline: non-smoker parents and high MHC). The measure of effect modification represents the size of the difference between the RD in CMs with (e.g.) low MHC and 1+ parent(s) who smoke compared with the RD for CMs with low MHC and no parent who smokes *plus* the RD for those who had high MHC and 1+ parent(s) who smoke. This second approach to measuring effect modification should be examined (alongside the first) when the effect modifier is a potential cause of the outcome, as is likely to be the case for MHC and CM smoking.^33^

A key assumption in effect modification analysis is that the exposure (in this case, parental smoking) is not a cause of the effect modifier (MHC).^34^ We posit that parental smoking does not influence the manifestation of skills represented by MHC.

### Imputation

Multiple imputations by chained equations were carried out, in 20 datasets, under a missing at random assumption. Estimates were combined using Rubin’s rules. The imputation model included exposure, outcome and confounder variables, MHC, variables used to account for sample design and attrition to age 14 y, and a number of auxiliary variables: whether anyone smoked around the child (at 7 y); Index of Multiple Deprivation (deciles), main language spoken in the household, household size, number of siblings and CM having tried a cigarette (all measured at 11 y); country of residence (at 9 months); and the main respondent’s age at CM birth. An interaction between MHC and parental smoking was included so that the imputation model was compatible with the effect modification analyses.

### Additional analyses

It is advised that results are reported on both additive and multiplicative scales,^33^ with the latter allowing readers to consider the potential impacts on relative differences maximizing comparability with other research. Therefore, we repeated analyses using risk ratios (RRs) (estimated in Poisson regression models, with robust standard errors) to consider effect modification on the multiplicative scale (Supplementary Appendix SA2).

Models were also repeated in a complete case sample (*n *=* *8693; Supplementary Appendix SA3).

## Results

### Characteristics of the sample

Table 1 shows the socio-demographic and health characteristics of the observed (any data recorded on a variable), complete case and main analytic (imputed) samples. The analytic sample was more disadvantaged and had higher proportions of ethnic minority groups. Thirty-five percent of CMs at 11 y had at least one parent who smoked. While the proportions of CMs who had ever smoked regular or e-cigarettes were similar (17 and 18%, respectively), the overlap between these two behaviours was moderate: 40% CMs who had ever smoked regular or e-cigarettes had smoked both. In other words, 9% CMs had smoked both regular and e-cigarettes at some point, with 6% smoking only cigarettes and 6% smoking only e-cigarettes.

**Table 1:** Characteristics of observed, complete case and analytic (imputed) samples

	MCS observed sample (*n* varies)		Complete case (*n *=* *8693)		Analytic (imputed) sample (*n *=* *10 133)
Characteristic	*N*	Weighted,^a^ %	*n*	Weighted,^b^ %	Weighted, %^b^
Parental smoking (age 11 y)					
Non-smoker parents	8794	65.1	6160	66.8	65.0
1+ smoker parents^c^	4223	34.9	2533	33.2	35.0
Not present at survey	*5868*				
Item missing	*95*				
Smoking in CM (age 14 y)					
Ever smoked cigarettes					
No	9397	83.0	7455	84.0	83.1
Yes	1606	17.0	1238	16.0	16.9
Not present at survey	*7404*				
Item missing	*573*				
Ever smoked e-cigarettes					
No	9324	82.3	7378	82.7	82.3
Yes	1684	17.7	1315	17.3	17.7
Not present at survey	*7404*				
Item missing	*568*				
MHC at age 11 y					
High MHC	4686	36.7	3488	37.5	36.4
High–Moderate MHC	4300	35.8	3132	36.4	36.1
Moderate MHC	2249	19.3	1585	19.0	19.0
Low MHC	847	8.2	488	7.0	8.5
Not present at survey	*5868*				
Item missing	*1030*				
Confounding variables					
CM sex					
Male	9775	51.4	4272	50.7	51.8
Female	9205	48.6	4421	49.3	48.2
Not present at survey	NA				
Item missing	*0*				
CM ethnicity					
White	15 237	85.7	7396	85.8	82.9
Other	3719	14.3	1297	14.2	17.1
Not present at survey	NA				
Item missing	*24*				
Mother’s highest academic qualifications (9 months)					
GCSE grade A*–C	12 503	71.8	6723	71.5	66.2
GCSE D–G and below	6336	28.2	1970	28.5	33.8
Not present at survey	NA				
Item missing	*141*				
Family structure (7 y)					
Natural parents	9859	69.7	6652	70.9	68.4
Reconstituted	891	7.7	492	7.3	7.9
Lone parent	2870	22.6	1549	21.8	23.7
Not present at survey	*5299*				
Item missing	*61*				
Mother’s psychological distress (7 y)					
No-low distress	9768	74.7	6711	75.1	73.0
Moderate/High distress	3223	25.3	1982	24.9	27.0
Not present at survey	*5299*				
Item missing	*690*				
CM socio-emotional problems, total SDQ score (7 y)					
Normal	11 377	86.3	7733	86.8	84.1
Borderline-abnormal	1812	13.7	960	13.2	15.9
Not present at survey	*5299*				
Item missing	*412*				
Annual household income, £s (11 y)					
Mean (SD)	£27 070	£349	£28 388	£342	£26 965 (£344)
Not present at survey	*5868*				
Item missing	*0*				

a: Weighted to account for sample design and attrition for the survey at which the measure was collected.

b: Weighted to account for sample design and attrition at the 14 y survey.

c: In the observed sample, 27% of mothers and 25% of partners smoked; in the complete case sample 25% mothers and 27% of partners smoked.

SDQ, strengths and difficulties questionnaire; GCSE, General Certificate in Secondary Education; CM, cohort member; SD, standard deviation. Missing data are highlighted in italics.

### Descriptive associations

CMs with lower levels of MHC were more likely to have a parent who smoked, as were those who were living in lone parent or lower-income households, whose mother had psychological distress or lower academic attainment, and who were of White ethnicity.

The prevalence of ever smoking cigarettes reported at 14 y was higher in CMs whose parent(s) smoked at 11 y (27.1%) than those whose parent(s) did not (11.4%) (table 2). Similar, but smaller, differences were observed for e-cigarettes, at 25.3% among those whose parent(s) smoked compared with 13.6% among those whose parent(s) did not smoke.

**Table 2:** Descriptive associations between parental smoking, CM smoking, MHC and confounding characteristics (*n *=* *10 133)

	Exposure	Outcomes	
Covariates	1+ parents smoked at 11 y (weighted %)	CMs ever smoked cigarettes at 14 y (weighted %)	CMs ever smoked e-cigarettes at 14 y (weighted %)
Exposure: parental smoking (age 11 y)			
Non-smoker parents		11.4	13.6
1+ parents who smoke		27.1	25.3
Effect modifier: MHC at the age of 11 y			
High MHC	27.4	11.7	13.9
High–Moderate MHC	38.5	19.3	19.4
Moderate MHC	36.5	16.5	18.0
Low MHC	49.5	29.8	26.2
Confounding variables			
CM sex			
Male	35.3	15.6	18.8
Female	34.7	18.3	16.5
CM ethnicity			
White	36.3	17.8	18.4
Other	28.7	12.7	14.2
Maternal academic qualification (at 9 months)			
GCSE grade A*–C and above	27.6	13.8	15.3
GCSE D–G and below	49.4	22.9	22.3
Family structure (7 y)			
Natural parents	29.0	12.7	14.6
Reconstituted	59.3	27.8	24.4
Lone parent	44.2	25.4	24.5
Mother’s psychological distress (7 y)			
No-low distress	31.4	15.2	16.4
Moderate–high distress	44.9	21.5	21.1
CM socio-emotional problems, total SDQ score (7 y)			
Normal	32.3	15.5	17.3
Borderline-abnormal	49.5	24.2	19.9
Household income, quintiles (11 y)			
Lowest quintile	57.1	24.8	23.5
2	44.2	21.1	20.8
3	29.6	14.6	17.3
4	20.4	10.5	12.7
Highest quintile	14.2	10.0	11.6

SDQ, strengths and difficulties questionnaire; GCSE, General Certificate in Secondary Education.

CMs with low MHC at the age of 11 y were the most likely to have ever smoked cigarettes (29.8%) and those with high MHC were the least likely (11.7%) (table 2). The prevalence of ever smoking cigarettes among those with Moderate and High–Moderate MHC were more similar to each other (16.5% and 19.3%, respectively). Comparable but weaker patterns were seen for e-cigarette use (ranging from 26.2% in low MHC to 13.9% in high MHC).

Girls were slightly more likely to have ever smoked cigarettes than boys, whereas the opposite was seen for e-cigarettes. White children, those with borderline-abnormal socio-emotional well-being, those living in reconstituted, lone parent or lower-income families, and those whose mothers had lower educational qualifications or experienced psychological distress were all more likely to have ever smoked cigarettes and e-cigarettes.

### Effect modification, Approach 1: association between parental and CM smoking, according to classes of MHC

An elevated risk of cigarette smoking in CMs whose parent(s) smoked was seen across all classes of MHC (table 3). However, the RD (or in other words, the number of CMs affected) was considerably larger for those with Low MHC [adjusted RD: 20.9% (11.2–30.6)] than those with high MHC [11.1% (7.1–15.1)] [measure of effect modification: 9.8% (−0.03, 19.9), thus indicating an interaction on the additive scale]. The effects of parental smoking on CM cigarette smoking were comparable in the Moderate–High [RD: 11.1% (7.3–14.9)] and High [11.1% (7.1–15.1)] MHC groups [measure of effect modification: 0.0% (−5.5, 5.6), indicating no additive interaction]. While the effects of parental smoking were lower in the moderate MHC class [7.0% (2.2–12.0)], there were wide confidence intervals around the measure of effect modification [4.0% (−10.0, 19.7)]. Patterns were similar for e-cigarette smoking, although they were less pronounced and with wide confidence intervals.

**Table 3:** Effect modification approach #1: RDs for CM cigarette and e-cigarette smoking according to parental smoking, within the MHC classes (*n *=* *10 133)

	High MHC	**High**–**Moderate MHC**	Moderate MHC	Low MHC
**Outcome: CM cigarette smoking**				
Prevalence (%)				
No parents smoke	7.9	13.6	12.6	18.3
1+ parents smoke	21.9	28.4	23.3	41.5
Risk differences (95% CI; *P*-values) according to parental smoking, within classes of MHC				
Unadjusted				
No parents who smoke	–	–	–	–
1+ parents smoke	14.0 (10.2, 17.9; <0.001)	14.8 (10.8, 18.8; <0.001)	10.7 (5.7, 15.6; <0.001)	23.3 (13.6, 32.9; <0.001)
Measure of effect modification (95% CI; *P*-values)		0.8 (−0.5, 6.2; 0.786)	−3.3 (−9.3, 2.6; 0.272)	9.2 (−1.1, 19.6; 0.081)
Adjusted^a^				
No parents who smoke	–	–	–	*–*
1+ parents smoke	11.1 (7.1, 15.1; <0.001)	11.1 (7.3, 14.9;<0.001)	7.0 (2.2, 12.0; 0.004)	20.9 (11.2, 30.6; <0.001)
Measure of effect modification (95% CI; *P*-values)		0.00 (−5.5, 5.6; 0.988)	−4.0 (−10.0, 19.7; 0.187)	9.8 (−0.03, 19.9; 0.058)
**Outcome: CM E-cigarette use**				
Prevalence (%)				
No parents smoke	11.1	14.6	15.3	19.2
1+ parents smoke	21.3	27.0	22.8	33.4%
Risk differences (95% CI; *P*-values), according to parental smoking, within classes of MHC				
Unadjusted				
No parents smoke	–	–	–	–
1+ parents smoke	10.2 (6.1, 14.2; <0.001)	12.4 (8.7, 16.1; <0.001)	7.5 (2.5, 12.5; 0.004)	14.2 (4.9, 23.5; 0.003)
Measure of effect modification (95% CI; *P*-values)		2.2 (−3.0, 7.5; 0.404)	−2.7 (−9.3, 3.8; 0.419)	4.0 (−6.3, 14.3; 0.443)
Adjusted^a^				
No parents smoke	–	–	–	–
1+ parents smoke	7.8 (3.6, 12.0; <0.001)	9.7 (6.0, 13.4; <0.001)	4.8 (−0.4, 9.9; 0.07)	12.4 (3.2, 21.6; 0.01)
Measure of effect modification (95% CI; *P*-values)		1.9 (−3.3, 7.1; 0.474)	−3.0 (−9.6, 3.5; 0.362)	4.6 (−5.5, 14.7; 0.369)

a: Adjusting for: CM’s ethnicity and sex, mother’s highest academic qualification and psychological distress, household income, family structure and child socio-emotional problems at the age of 7 y.

CI, confidence interval.

### Effect modification, Approach 2: risk of CM smoking according to combinations of parental smoking and MHC

RDs for CM ever smoking, according to the combination of parental smoking and class of MHC (baseline: no parents who smoked and High MHC, unadjusted prevalence 7.9%), are shown in table 4. CMs whose parent(s) smoked and who had low MHC were considerably more likely to have ever smoked than the baseline (CMs who had no parents who smoked and high MHC) [adjusted RD: 27.7% (20.0–35.5)]. This RD was greater than the sum of RDs for CMs with non-smoker parents and low MHC [6.9 (1.8–13.6)] and a smoker parent but high MHC [11.1 (7.1–15.1)] by 9.8% [−0.03, 19.9], as indicated by the measure of effect modification.

**Table 4:** Effect modification approach #2: RDs for CM cigarette and e-cigarette smoking, according to ‘combinations’ of MHC and parental smoking (*n *=* *10 133)

	High MHC	**High**–**Moderate MHC**	Moderate MHC	Low MHC
**Outcome: CM cigarette smoking**				
Prevalence (%)				
No parents smoke	7.9	13.6	12.6	18.3
1+ parents smoke	21.9	28.4	23.3	41.5
Risk differences (95% CI; *P*-values) according to combinations of parental smoking and MHC				
Unadjusted				
No parents who smoke	–	5.7 (3.6, 7.8; <0.001)	4.7 (2.2, 7.3; <0.001)	10.4 (4.0, 16.7; 0.001)
1+ parents smoke	14.0 (10.2, 17.9; <0.001)	20.5 (17.0, 24.0; <0.001)	15.4 (11.1, 19.8; <0.001)	33.7 (25.7, 41.6; <0.001)
Measure of effect modification (95% CI; *P*-values)		0.8 (−0.5, 6.2; 0.786)	−3.3 (−9.3, 2.6; 0.272)	9.2 (−1.1, 19.6; 0.081)
Adjusted^a^				
No parents who smoke	–	5.0 (2.9, 7.1; <0.001)	4.2 (1.6, 6.7; 0.002)	6.9 (1.8, 13.6; 0.04)
1+ parents smoke	11.1 (7.1, 15.1; <0.001)	16.1 (12.6, 19.6; <0.001)	11.2 (6.7, 15.7; <0.001)	27.7 (20.0, 35.5; <0.001)
Measure of effect modification (95% CI; *P*-values)		0.00 (−5.5, 5.6; 0.988)	−4.0 (−10.0, 19.7; 0.187)	9.8 (−0.03, 19.9; 0.058)
**Outcome: CM e-cigarette use**				
Prevalence (%)				
No parents smoke	11.1	14.6	15.3	19.2
1+ parents smoke	21.3	27.0	22.8	33.4
Risk differences (95% CI) according to combinations of parental smoking and MHC				
Unadjusted				
No parents who smoke	–	3.6 (1.1, 6.0; 0.004)	4.2 (1.1, 7.3; 0.01)	8.1 (1.9, 14.2; 0.01)
1+ parents smoke	10.2 (6.1, 14.2; <0.001)	16.0(12.3, 19.6; <0.001)	11.7 (7.0, 16.3;<0.001)	22.3 (14.4, 30.1; <0.001)
Measure of effect modification (95% CI; *P*-values)		2.2 (−3.0, 7.5; 0.404)	−2.7 (−9.3, 3.8; 0.419)	4.0 (−6.3, 14.3; 0.443)
Adjusted^a^				
No parents smoke	–	2.7 (0.02, 5.1; 0.04)	3.4 (0.02, 6.6; 0.04)	6.0 (−0.5, 12.4; 0.07)
1+ parents smoke	7.8 (3.6, 12.0; <0.001)	12.3 (8.7, 16.0; <0.001)	8.1 (3.2, 13.1; <0.001)	18.4 (10.0, 26.7; <0.001)
Measure of effect modification (95% CI; *P*-values)		1.9 (−3.3, 7.1; 0.474)	−3.0 (−9.6, 3.5; 0.362)	4.6 (−5.5, 14.7; 0.369)

a: Adjusting for: CM’s ethnicity and sex, mother’s highest academic qualification and psychological distress, household income, family structure and child socio-emotional problems at the age of 7 y.

CI, confidence interval.

There was little evidence of additive interaction between parental smoking and Moderate or High–Moderate MHC; that is, the combined effects of parental smoking and Moderate/Moderate–High MHC was no greater than the sum of the individual effects of only having a parent who smoked and only having Moderate/Moderate–High MHC. This pattern was apparent but weaker for e-cigarette use with wide confidence intervals.

As expected, this second approach produced the same conclusions as to the first; that Low (but not Moderate or High–Moderate) MHC potentially amplified the adverse effects of parent smoking on CM smoking behaviours.

### Sensitivity analyses

The main results examined effect modification on the additive scale (looking at absolute differences between RDs) since these are of greater relevance to public health.^33^ As recommended,^33^ we repeated the analyses using RRs (Supplementary Appendix table SA1), to examine effect modification on the multiplicative scale (i.e. relative differences between RRs). There was no evidence of an excess risk due to multiplicative interaction among those with low MHC. However, there was a reduced risk among those with Moderate or High–Moderate MHC (further details are provided in Supplementary Appendix S2). Therefore, interventions to improve MHC hold the potential to reduce absolute inequalities, but widen relative inequalities, in the prevalence of smoking initiation between those whose parents do and do not smoke.

Effect modification analyses were also repeated in the complete case sample and patterns were similar to the imputed results reported (Supplementary Appendix tables SA2 and SA3).

## Discussion

### Summary of findings

In a UK-representative, contemporary cohort, we have shown that young people who had at least one parent who smoked when they were 11 y were considerably more likely to have ever smoked cigarettes by 14 y. Using two complementary approaches for testing effect modification, results on the additive scale indicated that the influence of parental smoking behaviours on CM smoking of traditional cigarettes was especially strong for CMs with Low (but not Moderate or High–Moderate) MHC, compared with those with High MHC. While the patterning of results for e-cigarettes was similar, the degree of modification was considerably smaller and did not reach statistical significance. These associations were robust to adjustment for confounders and were similar in the analytic (imputed) sample and complete case analyses. Findings suggest that improving low MHC, even to moderate levels, may have the potential to reduce the number of youth smoking initiation cases associated with parental smoking, which is a prominent risk factor for the uptake of smoking.

### Comparison with other findings

A strong association between parent and offspring smoking has been established in a worldwide systematic review and meta-analysis of more than 50 studies^10^ and in subsequent research using large, nationally representative datasets in the USA^35^ and the UK.^36^ However, there is a paucity of research that has investigated factors that might reduce the likelihood of teenagers taking up smoking when exposed to important risk factors,^37^ such as having parents who smoke. The possible protective influence of higher MHC on adolescent cigarette smoking has previously been observed in the MCS^19^ and research from elsewhere has also demonstrated that measures reflecting particular MHC skills in childhood or early adolescence, such as greater self-control^21^; emotional control, inhibitory control, working memory and planning^22^; and cognitive abilities^20^ are associated with reduced risk of smoking in adolescence and adulthood. Few studies have examined factors that may alter the relationship between parent and child smoking behaviours. One study from the Philippines found that peer support protected against the transmission of smoking behaviours between parents and children, while anxiety levels exacerbated this relationship.^38^ In this current study, we show, for the first time, that increasing MHC at the end of primary school may not only support young people to resist taking up smoking but particularly protect those at greater risk due to parental smoking. This finding is notable since social and emotional learning programmes in schools have been demonstrated to improve aspects of MHC,^39^ and these can be implemented before health-risk behaviours, such as smoking, are often initiated. Such interventions may carry other beneficial effects, for example, for academic outcomes and physical health.^18^^,^^25^^,^^26^

### Strengths and limitations

We examined the potential for MHC in childhood to buffer against the inter-generational transmission of smoking behaviours using a large, contemporary UK-wide cohort. We were able to examine two outcomes (cigarette and e-cigarette smoking), the smoking behaviours of resident parents, and to adjust for potential confounders. Weights were used to adjust for attrition to the 14 y survey, and multiple imputations accounted for item missingness. Findings in the complete case sample were similar to those of the imputed sample.

We were not able to identify whether parents who smoked did so inside or outside the home, or their degree of nicotine dependency. Furthermore, we were only able to explore smoking initiation and not longer-term smoking behaviours, because of the relatively young age of the CMs. Future work should examine how MHC relates to regular smoking behaviours in young adulthood. Smoking behaviours of parents and CMs were drawn from self-report responses to questions answered using hand-held devices, with confidentiality emphasized. This reduced, although did not eliminate, the potential for social desirability report bias.^40^ The survey questions on smoking behaviours prevented us from assessing tobacco consumption other than cigarettes, although we would expect use of such products to be of very low prevalence. MHC was based on maternal report items from the SDQ, which although validated (as a total score and individual scales), may be susceptible to response bias. We adjusted for a range of potential confounding factors, but it remains possible that CMs with Low MHC differ from their peers in ways that we have not been able to account for, and that this confounds the stronger association between parent and smoking in this group. Finally, MHC did not modify the association between parent and child smoking behaviours in a dose–response manner. Future research should seek to replicate these findings at other ages and in different datasets.

### Implications for policy, practice and further research

Our findings provide evidence that improving the MHC of those young people with low levels may reduce the number that start smoking among those whose parents are smokers more so than those who do not. Since an association between parent and offspring smoking is observed the world over, we posit that the findings from the present analysis have potential international relevance. MHC, as operationalized here, can be captured with items from existing measures, such as the SDQ^19^ and the Early Development Index,^17^ which are commonly included in surveys and school censuses around the globe. Our analyses might therefore be replicated and extended using data recorded in other populations, or with MHC and exposures or outcomes measured at different stages of childhood and adolescence. Future research should also examine whether the benefits of higher MHC for health behaviours demonstrated here can be replicated in randomized controlled trials. Interventions that improve MHC, even to moderate levels, may hold potential for reducing the public health burden of smoking due to intergenerational transference.

## Supplementary data

Supplementary data are available at *EURPUB* online.

## Supplementary Material

ckab102_Supplementary_Data
