# Supplementary material for: Can mental health competence reduce the higher risk of smoking initiation among teenagers with parents who smoke?
Source: Eur J Public Health. 2021 Sep 18;31(4):756–63. doi: 10.1093/eurpub/ckab102 (PMC8561261; doi:10.1093/eurpub/ckab102)
Supplement: ckab102_Supplementary_Data [file ckab102_Supplementary_Data.docx]

**Appendix 1**

*Figure A1: Hypothesised relationship between variables of interest*


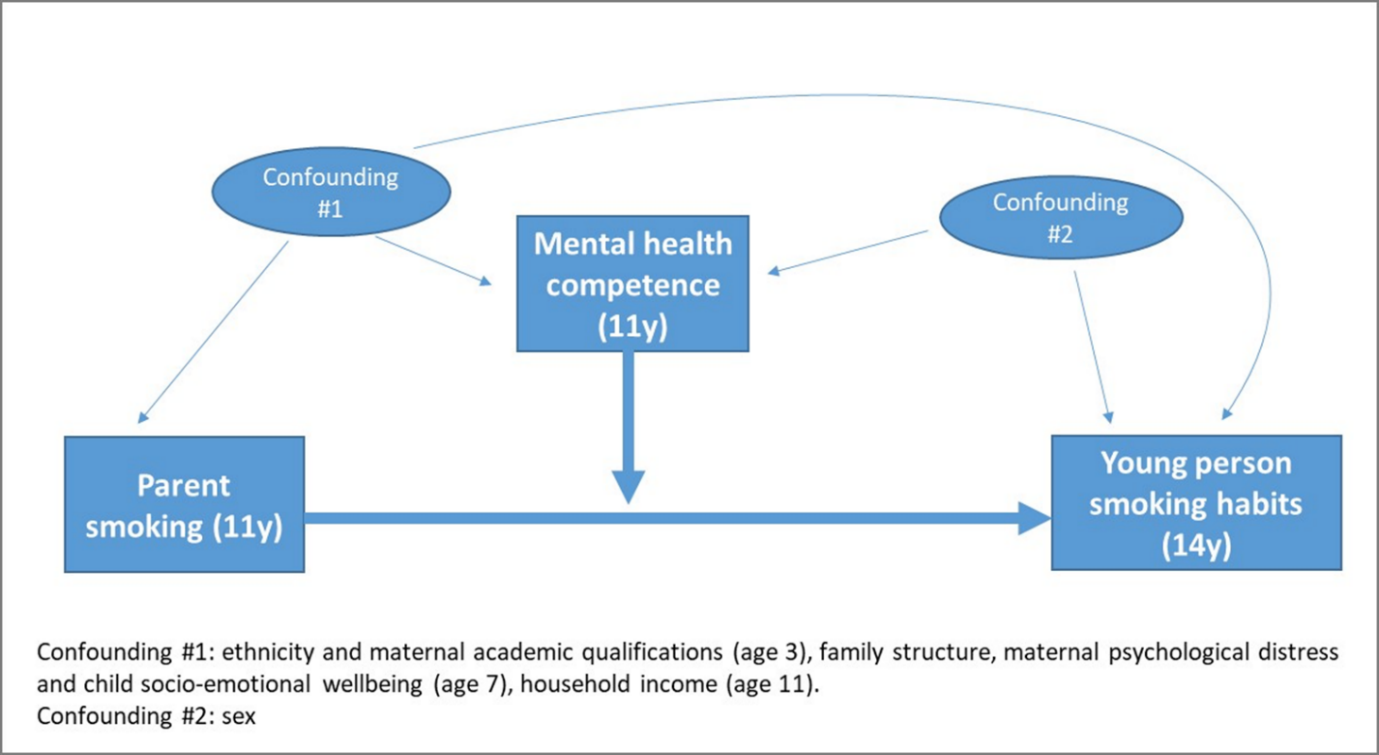


**Appendix 2: Effect measure modification on the multiplicative scale (using relative risks)**

Additive interactions are relevant to our research question, focusing on public health implications for adolescent smoking of changes in MHC. Here we present interactions on the multiplicative scale, as recommended by Knol et al(1), for readers who are interested in relative as opposed to absolute differences.

Table A1 presents results for the two approaches to effect modification (on the multiplicative scale) for CM smoking and e-cigarettes, using risk ratios (RR), before and after adjustment for confounding.

Approach 1: Although an association between CM and parent smoking was seen across all levels of MHC, RRs were largest in the High and Low MHC classes and lowest in the High-Moderate and Moderate MHC classes. All results were similar for e-cigarette use.

Approach 2: The highest RR for smoking was observed in CMs with Low MHC *and* at least one parent who smoked: 3.79 (2.88, 4.99), baseline: High MHC and no parents who smoked). However, this was slightly lower than the sum of the RRs in those who had Low MHC but no parent who smoked (1.88 (1.27, 2.80)), and in CMs who had at least one parent who smoked but High MHC (2.31 (1.81, 2.95)), although was not statistically significant. Similar, but slightly stronger, patterns were seen for Moderate or High-Moderate MHC. Results were similar for e-cigarette use.

If results for effect modification on absolute and relative scales are considered together, they suggest that interventions to improve MHC hold the potential to reduce absolute differences, but increase relative differences, in youth smoking between those whose parent(s) do versus those whose parent(s) do not smoke.

*Table A1: Effect modification analyses examining the association between CM and parent smoking according to MHC, using risk ratios (95% CIs; p-values). n=10133*

|  | **High MHC** | **High-Moderate MHC** | **Moderate MHC** | **Low MHC** |
| --- | --- | --- | --- | --- |
| *Outcome: Young person Cigarette smoking* | | | | |
| **Effect modification approach #1: Risk ratios according to parent smoking, within levels of MHC** | | | | |
| *Unadjusted* | | | | |
| No parents who smoke | *-* | *-* | *-* | *-* |
| 1+ parents smoke | 2.78 (2.20, 3.53; <0.001) | 2.09 (1.74, 2.51; <0.001) | 1.85 (1.43, 2.39; <0.001) | 2.28 (1.54, 3.37; <0.001) |
| Measure of effect modification (95% CI; p-values) | | 0.75 (0.56, 1.300; 0.052) | 0.66 (0.48, 0.92; 0.015) | 0.82 (0.53, 1.26; 0.363) |
| *Adjusted** | | | | |
| No parents who smoke | *-* | *-* | *-* | *-* |
| 1+ parents smoke | 2.31 (1.80, 2.95; <0.001) | 1.69 (1.41, 2.02; <0.001) | 1.49 (1.14, 1.94; 0.004) | 2.01 (1.37, 2.96; <0.001) |
| Measure of effect modification (95% CI; p-values) | | 0.73 (0.55, 0.98; 0.034) | 0.64 (0.46, 0.89; 0.009) | 0.87 (0.57, 1.34; 0.527) |
| **Effect modification approach #2: Risk ratios according to combinations of parent smoking and MHC** | | | | |
| *Unadjusted* | | | | |
| No parents who smoke | - | 1.72 (1.41, 3.53; <0.001) | 1.60 (1.27, 2.19; <0.001) | 2.31 (1.59, 3.37; <0.001) |
| 1+ parents smoke | 2.78 (2.20, 3.53; <0.001) | 3.60 (2.95, 4.41; <0.001) | 2.96 (2.32, 3.77; <0.001) | 5.27 (4.05, 6.85; <0.001) |
| Measure of effect modification (95% CI; p-values) | | 0.75 (0.56, 1.300; 0.052) | 0.66 (0.48, 0.92; 0.015) | 0.82 (0.53, 1.26; 0.363) |
| *Adjusted** | | | | |
| No parents who smoke | - | 1.64 (1.35, 2.00) | 1.54 (1.22, 1.93) | 1.88 (1.27, 2.80) |
| 1+ parents smoke | 2.31 (1.81, 2.95) | 2.77 (2.26, 3.41) | 2.29 (1.78, 2.96) | 3.79 (2.88, 4.99) |
| Measure of effect modification (95% CI; p-values) | | 0.73 (0.55, 0.98; 0.034) | 0.64 (0.46, 0.89; 0.009) | 0.87 (0.57, 1.34; 0.527) |
| *Outcome: Young person E-cigarette consumption* | | | | |
| **Effect modification approach #1: Risk ratios according to parent smoking, within levels of MHC** | | | | |
| *Unadjusted* | | | | |
| No parents who smoke | - | - | - | - |
| 1+ parents smoke | 1.92 (1.53, 2.41; <0.001) | 1.85 (1.56, 2.19; <0.001) | 1.49 (1.15, 1.92; 0.02) | 1.74 (1.20, 2.53; 0.04) |
| Measure of effect modification (95% CI; p-values) | | 0.96 (0.73, 1.27; 0.787) | 0.78 (0.55, 1.09; 0.149) | 0.91 (0.58, 1.41; 0.668) |
| *Adjusted** | | | | |
| No parents who smoke | - | - | - | - |
| 1+ parents smoke | 1.67 (1.31, 2.11; <0.001) | 1.58 (1.33, 1.88; <0.001) | 1.28 (0.97, 1.66; 0.071) | 1.57 (1.10, 2.26; 0.01) |
| Measure of effect modification (95% CI; p-values) | | 0.95 (0.72, 1.25; 0.705) | 0.77 (0.54, 1.08; 0.124) | 0.95 (0.61, 1.45; 0.797) |
| **Effect modification approach #2: Risk ratios according to combinations of parent smoking and MHC** | | | | |
| *Unadjusted* | | | | |
| No parents who smoke | - | 1.32 (1.09, 1.60; 0.004) | 1.38 (1.10, 1.72; 0.005) | 1.73 (1.22, 2.44; 0.002) |
| 1+ parents smoke | 1.920 (1.53, 2.41; <0.001) | 2.44 (2.02, 2.94; <0.001) | 2.05 (1.61, 2.62; <0.001) | 3.00 (2.28, 3.95; <0.001) |
| Measure of effect modification (95% CI; p-values) | | 0.96 (0.73, 1.27; 0.787) | 0.78 (0.55, 1.09; 0.149) | 0.91 (0.58, 1.41; 0.668) |
| *Adjusted** | | | | |
| No parents who smoke | - | 1.25 (1.03, 1.52; 0.02) | 1.31 (1.03, 1.52; 0.02) | 1.52 (1.06, 2.17; 0.02) |
| 1+ parents smoke | 1.67 (1.31, 2.11; <0.001) | 1.98 (1.62, 2.41; <0.001) | 1.67 (1.28, 2.17; <0.001) | 2.39 (1.74, 3.28; <0.001) |
| Measure of effect modification (95% CI; p-values) | | 0.95 (0.72, 1.25; 0.705) | 0.77 (0.54, 1.08; 0.124) | 0.95 (0.61, 1.45; 0.797) |

*Adjusting for: CM’s ethnicity, sex and socio-emotional problems (age 7), mother’s highest academic qualification and psychological wellbeing, household income and family structure.

**Appendix 3: Results from complete case analysis**

*Table A2: Effect modification approach #1: Risk Differences (RDs) for cohort member (CM) cigarette and e-cigarette smoking according to parental smoking, stratified by mental health competence (MHC). n=8,693*

|  | **High MHC** | **High-Moderate MHC** | **Moderate MHC** | **Low MHC** |
| --- | --- | --- | --- | --- |
| **Outcome: CM Cigarette smoking** | | | | |
| **Prevalence** | | | | |
| No parents smoke | 7.5% | 13.6% | 12.7% | 17.9% |
| 1+ parents smoke | 21.1% | 26.4% | 25.5% | 39.4% |
| **Risk differences (95% CI) according to parental smoking, within levels of MHC** | | | | |
| *Unadjusted* | | | | |
| No parents who smoke | - | - | - | - |
| 1+ parents smoke | 13.6 (9.6, 17.5) | 12.8 (9.0, 16.6) | 12.8 (7.5, 18.1) | 21.5 (10.6, 32.4) |
| Measure of effect modification (CIs; p value) | | -0.7% (-6.1, 4.6) | -0.8 (-7.2, 5.7) | 8.0 (-3.5, 19.4) |
| *Adjusted** | | | | |
| No parents who smoke | *-* | *-* | *-* | *-* |
| 1+ parents smoke | 10.9 (6.9, 14.9) | 9.4 (5.6, 13.2) | 8.9 (3.5, 14.4) | 19.0 (8.3, 29.7) |
| Measure of effect modification (CIs; p value) | | -1.5 (-6.9, 3.8) | -2.0 (-8.5, 4.5) | 8.1 (-3.1, 19.3) |
| **Outcome: CM E-cigarette use** | | | | |
| **Prevalence** | | | | |
| No parents smoke | 11.1% | 14.7% | 15.3% | 18.0% |
| 1+ parents smoke | 20.7% | 25.1% | 25.3% | 35.8% |
| **Risk differences (95% CI), according to parental smoking, within levels of MHC** | | | | |
| *Unadjusted* | | | | |
| No parents smoke | - | - | - | - |
| 1+ parents smoke | 9.6 (5.4, 13.7) | 10.4 (6.7, 14.1) | 10.0 (4.5, 15.5) | 17.9 (7.14, 28.6) |
| Measure of effect modification (CIs; p value) | | 0.8 (-4.6, 6.2) | 0.4 (6.6, 7.5) | 8.3 (-3.1, 19.7) |
| *Adjusted** | | | | |
| No parents smoke | - | - | - | - |
| 1+ parents smoke | 7.5 (3.1, 11.9) | 8.0 (4.1, 11.8) | 7.2 (1.5, 12.8) | 16.2 (5.7, 26.8) |
| Measure of effect modification (CIs; p value) | | 0.5 (-4.9, 5.9) | -0.3 (-7.4, 6.7) | 8.7 (-2.4, 19.8) |

*Adjusting for: CM’s ethnicity, sex and socio-emotional problems (age 7), mother’s highest academic qualification and psychological wellbeing, household income and family structure.

*Table A3: Effect modification approach #2: Risk Differences (RDs) for cohort member (CM) cigarette and e-cigarette smoking, according to combinations of mental health competence (MHC) and parental smoking. n=8,693*

|  | **High MHC** | **High-Moderate MHC** | **Moderate MHC** | **Low MHC** |
| --- | --- | --- | --- | --- |
|  | **Outcome: CM Cigarette smoking** | | | |
|  | **Prevalence** | | | |
| No parents smoke | 7.5% | 13.6% | 12.7% | 17.9% |
| 1+ parents smoke | 21.1% | 26.4% | 25.5% | 39.4% |
|  | **Risk differences (95% CI) according to combinations of parental smoking and MHC** | | | |
|  | *Unadjusted* | | | |
| No parents who smoke | - | 6.1 (3.9, 8.2) | 5.1 (2.6, 7.5) | 10.3 (3.6, 17.1) |
| 1+ parents smoke | 13.6 (9.6, 17.5) | 18.9 (15.4, 22.4) | 17.9 (13.1, 22.8) | 31.9 (22.8, 40.9) |
| Measure of effect modification (CIs; p value) | | 1.1% (-3.9, 6.0) | -2.3 (-8.0, 3.4) | 8.8 (-5.9, 18.2) |
|  | *Adjusted** | | | |
| No parents who smoke | - | 5.5 (3.5, 7.6) | 5.0 (2.5, 7.5) | 7.3 (0.2, 14.9) |
| 1+ parents smoke | 10.9 (6.9, 14.9) | 14.9 (11.3, 18.5) | 13.9 (8.9, 19.0) | 26.3 (17.5, 35.1) |
| Measure of effect modification (CIs; p value) | | -1.5 (-6.9, 3.8) | -2.0 (-8.5, 4.5) | 8.1 (-3.1, 19.3) |
|  | **Outcome: CM E-cigarette use** | | | |
|  | **Prevalence** | | | |
| No parents smoke | 11.1% | 14.7% | 15.3% | 18.0% |
| 1+ parents smoke | 20.7% | 25.1% | 25.3% | 35.8% |
|  | **Risk differences (95% CI) according to combinations of parental smoking and MHC** | | | |
|  | *Unadjusted* | | | |
| No parents who smoke | - | 3.6 (0.9, 6.3) | 4.2 (1.0, 7.4) | 6.8 (-0.1, 14.5) |
| 1+ parents smoke | 9.6 (5.4, 13.7) | 14.0 (10.3, 17.7) | 14.2 (9.1, 19.4) | 24.7 (15.8, 33.7) |
| Measure of effect modification (CIs; p value) | | 0.8 (-4.6, 6.2) | 0.4 (6.6, 7.5) | 8.3 (-3.1, 19.7) |
|  | *Adjusted** | | | |
| No parents smoke | - | 2.9 (0.1, 5.6) | 3.6 (0.3, 6.9) | 5.3 (0.3, 13.1) |
| 1+ parents smoke | 7.5 (3.1, 11.9) | 10.9 (7.0, 14.7) | 10.8 (5.3, 16.3) | 21.6 (12.5, 30.7) |
| Measure of effect modification (CIs; p value) | | 0.5 (-4.9, 5.9) | -0.3 (-7.4, 6.7) | 8.7 (-2.4, 19.8) |

*Adjusting for: CM’s ethnicity, sex and socio-emotional problems (age 7), mother’s highest academic qualification and psychological wellbeing, household income and family structure.
